# Fussy Eating Rescue, a mobile-web app for responsive feeding practises among parents of toddlers: protocol for a pilot randomised controlled feasibility trial

**DOI:** 10.1186/s40814-023-01278-2

**Published:** 2023-07-22

**Authors:** Brittany Reese Markides, Kylie D. Hesketh, Ralph Maddison, Rachel Laws, Elizabeth Denney-Wilson, Karen J. Campbell

**Affiliations:** 1 Institute for Physical Activity and Nutrition (IPAN), School of Exercise and Nutrition Sciences, Deakin University Burwood, Victoria, Australia; 2 Susan Wakil School of Nursing and Midwifery, The University of Sydney, Camperdown, NSW Australia

**Keywords:** Parents, Child, Preschool, Infant, Diet, food and nutrition, Feeding-related behaviour, Digital health interventions, mHealth

## Abstract

**Background:**

Fussy eating is most often a developmentally typical behaviour, generally presenting during toddlerhood. However, up to half of parents of young children are concerned about fussy eating, and this concern may mediate the use of nonresponsive feeding practises, such as coercive or unstructured feeding and using food to reward eating. Despite the high prevalence of parental concern for fussy eating and the negative impacts nonresponsive feeding practises have on children’s health and diets, no previous digital intervention to improve the feeding practises of parents of toddlers concerned about fussy eating has been evaluated.

**Aim:**

This article describes the protocol of a randomised controlled feasibility pilot aiming to evaluate Fussy Eating Rescue, a purely web app based intervention for parents of toddlers. The primary aim is to investigate feasibility and acceptability; secondary aims are to explore indications of intervention effect on parents’ feeding practises or children’s eating behaviours.

**Methods:**

Fussy Eating Rescue features include: (1) a Tracker, that allows parents to track repeated offers of food, (2) Topics, providing information on fussy eating, effective feeding strategies, and general nutrition, (3) Rescues, containing quick references to material supporting Topics contents, (4) Recipes, and (5) SMS notifications. Parents of toddlers (12–36 months old, *n* = 50) who have concerns about fussy eating will be recruited via Facebook. Parents will be randomised to an intervention group, which receives access to the app for 6 weeks, or to wait-listed control. Outcomes will be assessed at baseline and 6 weeks after app use, using online questionnaires and app usage statistics. Primary outcomes include participant retention rate, intervention engagement, app usability, perceived ease in using the app, perceived usefulness of the app, and user satisfaction. Secondary outcome measures include parents’ feeding practises and children’s eating behaviours.

**Discussion:**

Results will inform whether Fussy Eating Rescue is a feasible way to engage parents concerned for their toddler’s fussy eating behaviours. If feasible and acceptable to users, a larger trial will further examine the efficacy of the Fussy Eating app in improving parents’ feeding practises and children’s eating behaviours.

**Trial registration:**

Prospectively registered with the Australian New Zealand Clinical Trials Registry on 15 July, 2021 (ACTRN12621000925842).

## Background

The parent–child feeding relationship is fundamental to early childhood development [1]. Parent–child feeding interactions have important bonding, socialization, and learning opportunities for children and are a key influence of young children’s diet quality [2]. Responsive feeding practises that support children’s autonomy to eat in response to their development and physiological needs have been shown to have positive impacts on children’s diet and health [3, 4]. In contrast, nonresponsive feeding practises—such as coercive feeding (e.g. pressuring to eat, coercive rules), unstructured feeding (e.g. allowing child to graze during the day), and using food to reward eating—have negative impacts on children’s’ ability to attend to internal signals of hunger and satiety and contribute to poor dietary outcomes and the development of long-term unhealthy eating preferences, i.e. low intakes of nutrient-dense foods (e.g. vegetables, fruits, whole grains) and high intakes of discretionary foods (e.g. savoury and sweet snacks, desserts, sugar-sweetened beverages) [3, 4]. In the long-term, poor diets are associated with the development of non-communicable diseases, including diabetes and heart disease, stroke and cancer, hypertension, non-alcoholic fatty liver disease, and poor bone health [5]. Previous longitudinal and cross-sectional research suggests that nonresponsive feeding practices are commonly used by parents who describe their child as a “fussy” or “picky” eater [6–13]. Fussy eating is variably defined as eating a limited variety or amount of food, having strong food preferences, or rejecting foods that are unfamiliar, have specific textures, or are in particular groups or categories [14]. While the lack of a clear definition for fussy eating makes estimating its prevalence problematic [15–17], studies have reported that up to 50% of parents of children 2–5 years are concerned about fussy eating [9, 18–20] and parents concern for fussy eating is a primary mediator of parents’ use of nonresponsive feeding practices [9, 18–20].

Fussy eating most commonly begins in early toddlerhood (12 to 24 months) and peaks in intensity in later toddlerhood (24 to 36 months) [21, 22]. The evidence suggests that most toddlers described as ‘fussy eaters’ by their parents are likely exhibiting developmentally typical eating behaviours that are likely to resolve as children age [20]. However, nonresponsive feeding practises may increase the intensity of and duration of fussy eating behaviours [9–12]. Therefore, targeted interventions that addresses parents’ concerns for their toddlers’ fussy eating behaviours are warranted.

Parents of young children have expressed a preference to engage digitally with health promotion content [23, 24]. An important advantage of digital health interventions is that they allow participants to access content at any time and any place of their choosing, which may mitigate barriers related to transportation, childcare, and scheduling conflicts [25–27]. Using digital platforms also provides opportunities to tailor content so that it is perceived to be personally relevant to the user [28]. Further, digital health interventions may also have advantages related to cost [29, 30] and scalability [25].

Previous digital parent feeding interventions have shown promise in improving parents feeding practices [31, 32]; however, no digital intervention has been developed to improve the feeding practises specifically among parents of toddlers concerned for fussy eating. Given the high proportion of parents concerned about fussy eating [8, 18–20], evidence that underlying fussy eating may undermine child nutrition interventions [33], and the general need for effective, scalable parent-centred feeding interventions [34], the development of successful interventions among this population is warranted.

The primary aim of this study is to assess the acceptability and feasibility of Fussy Eating Rescue, a fully-automated web app that aims to improve the feeding practises of parents concerned for toddler fussy eating behaviours. Primary feasibility measures include assessing participant retention rate, intervention engagement, app usability, perceived ease in using the app, perceived usefulness of the app, and user satisfaction. A secondary aim is to explore any indications of intervention effect.

## Methods

### Design

**Fig. 1:**
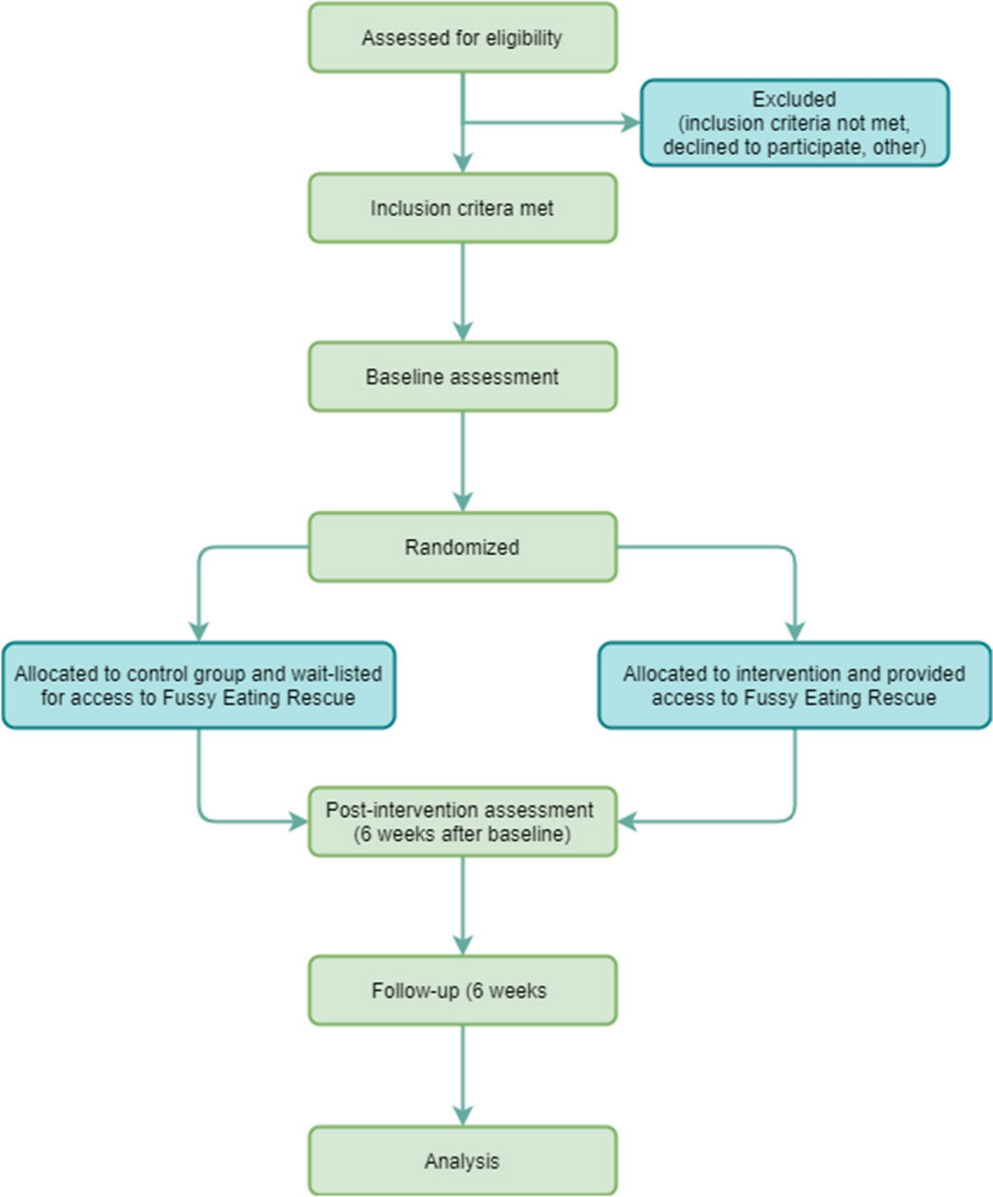
CONSORT diagram for the randomised controlled pilot study evaluating the feasibility of Fussy Eating Rescue

This protocol describes a 6-week, two-arm, parallel, randomised control pilot trial to evaluate a purely web app based intervention to improve the feeding practises of parents of toddlers (12–36 months) who are concerned for their toddler’s fussy eating behaviours. Parents assigned to the intervention group will have access to the web app containing information, strategies, recipes, and activities to track feeding attempts. Parents in the intervention group will also be able to opt into receiving to 42 SMS messages across the 6 weeks with tips and prompts to revisit the app. Pre and post-measures collected from a control group of parents (placed on a six week waitlist) will be used to evaluate indications of intervention effect. The description of the protocol and intervention are in line with the CONSORT guidelines for randomised pilot and feasibility trials [35]. A CONSORT diagram is provided in \* MERGEFORMAT Fig. 1 and the CONSORT checklist. The trial is prospectively registered at the Australian New Zealand Clinical Trials Registry (ACTRN12621000925842) and ethics approval was obtained from Deakin University Human Research Ethics Committees (2021–139).

### Study sample and recruitment

Parents who self-identify as concerned for the fussy eating behaviours of their toddler (12–36 months old) will be recruited via paid Facebook advertisements. The Facebook advertisement audience settings will be used to target adults (18–45 years) living in Australia who have general interests related to parenting toddlers. To address a common issue of study recruitment involving the overrepresentation of participants with relatively high socioeconomic position, a geographic location parameter will also be used to preferentially show advertisements to Facebook users who live in postcodes in the lower 2 deciles of the Socio-Economic Indexes for Areas (SEIFA), a product developed by the Australian Bureau of Statistics that ranks Australian areas based on measures of relative socio-economic advantage and disadvantage [37]. Participants will be eligible for inclusion if they currently live in Australia, are able to read and write in English, have a mobile phone that can access the internet and care for a child who is 12–36 months old and lives with them at least half of the time. The intervention has been developed to address the concerns of parents whose children display developmentally typical eating behaviours and have no co-existing conditions that present additional nutritional risk. Given this, participants will be excluded if they indicate that their child has been diagnosed with autism spectrum disorder, learning difficulties, avoidant/restrictive intake disorder, or a medical condition that requires them to follow special dietary requirements (e.g. type 1 diabetes, cystic fibrosis, celiac disease, or metabolic conditions).

Individuals who click on the Facebook ads will be taken to a REDCap screening survey hosted at Deakin University [38]. Eligible participants will be provided with a written introduction to the study and a plain language statement outlining the study protocols before providing informed consent.

Informed by evidence from the literature on sample size requirements for pilot RCTs, it is estimated that a sample of 40 participants will generate sufficient data to determine the feasibility of the intervention [39, 40]. To allow for a potential 20% attrition rate, a total of 50 participants will be recruited.

Participants will be randomised to either the Fussy Eating intervention or a wait-listed control group (25 participants in each group). The allocation sequence generation will be completed via the REDCap randomization module. Due to the wait-list design, participants will be aware of whether or not they are receiving an intervention, and this study will be non-blinded.

Intervention.

### *I*ntervention content

#### App development overview

**Fig. 2:**
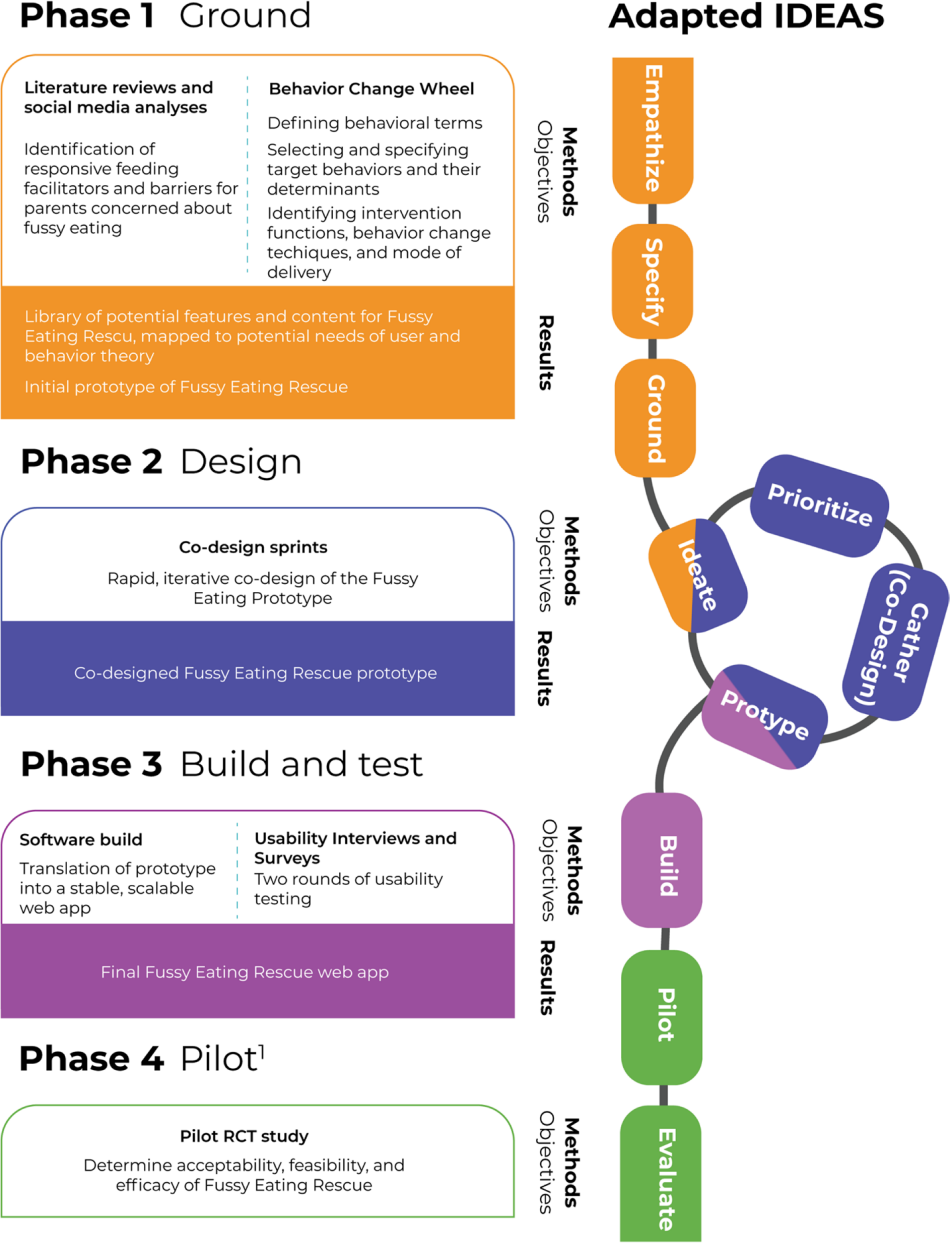
Development phases for Fussy Eating Rescue as it aligns with the IDEAS (Integrate, Design, Assess, and Share) frame work [36], adapted for iterative co-design activities

Development was completed in three phases: (1) Ground, (2) Design, (3) Build and Test. This paper describes the protocol for the final phase: (4) Pilot (Fig. 2).

#### Grounding in theory and previous research

A systematic, evidence-based approach was used to develop the Fussy Eating Rescue mHealth intervention using the Theoretical Domains Framework [41], Behavior Change Wheel (BCW) [42], and Behavior Change Techniques Taxonomy v1 (BCTTv1) [43]. This process was informed by researchers’ formative qualitative studies among the intended users of *Fussy Eating Rescue* [44] and consultation of the existing literature related to fussy eating and parent feeding practices in the toddler years [45]. Per the BCW, the researchers first identified the problem in behavioural terms, i.e. that parents concerned their toddler’s fussy eating behaviours were using nonresponsive feeding practices. The target behaviours were feeding practices identified in the literature as helpful versus problematic in the context of fussy eating. Responsive behaviours to promote included: repeated exposure (exposing children repeatedly to a wide variety of healthful foods, even those they have previously refused) [22, 46, 47], family meals [48], role modelling [49, 50], and meal and snack routines [51, 52]. Nonresponsive practices to discourage included pressure [11, 53], catering (offering children alternative foods when initial foods are rejected or offering a limited number of foods per child’s current taste preferences [7, 8, 11, 51, 54], and using food to reward eating or good behavior [7, 11].

Qualitative research previously undertaken by the research team [44, 55] and studies highlighted by a recent systematic review and synthesis of previous qualitative studies examining fussy eating [45] were consulted to compile a list of barriers and facilitators to using responsive feeding practices. Facilitators included: self-efficacy beliefs [44, 45], focusing on long-term outcome goals for their child’s eating behaviours [44] ability to self-regulate emotions and behaviours [44], knowledge that fussy eating is common and developmentally normal [44, 45, 55] knowledge of how children develop food preferences [44, 45, 55], and knowledge of what feeding practices are effective and ineffective [44, 45, 55]. Barriers included: parent concern about fussy eating (e.g. inadequate nutrient/energy intake, food waste, child going hungry, behavioural issues related to food refusal) [45, 48, 56], distrust in children’s hunger regulation [45], and attributing fussy eating to behavioural issues [45].

#### Human-centred design framework

App design took a human-centered approach, guided by the IDEAS (Integrate, Design, Assess, and Share) Framework [36]. IDEAS is a design framework and toolkit for the human-centred development of digital behavior change interventions that can be used to engage participants throughout the intervention development and is composed of 10 phases of design (empathise, specify, ground, ideate, prototype, gather, build, pilot, evaluate, and share), grouped into 4 stages: Integrate, Design, Assess, and Share (IDEAS). To develop features and content, parents and researchers worked together via video conferencing interviews to develop and evaluate a series of prototype features and content that may help parents adopt child-centred responsive feeding practises and discourage the use of authoritarian-style parent feeding practises, such as persuasive/coercive feeding. Primary features include (1) a Tracker that allows parents to keep track of the times they’ve offered specific foods to their child and to track their child’s response, (2) topics that provide information on why toddler fussy eating behaviour is normal, child-centred feeding strategies to adopt, and how much food toddlers need, (3) rescues, which are quick references to material that support the Topics contents (e.g. lists of non-food rewards, tips for managing food-related tantrums, ideas for addressing food waste concerns), and (4) recipes (a variety of healthful, simple recipes with a variety of vegetable ingredients were sourced from the My Baby Now app [57] and adapted to be age-appropriate for toddlers).

#### Intervention delivery

Participants in the intervention group will be sent an email with instructions for how to access the intervention app to their personal smartphone. The email will contain a link to the webpage where the web app is hosted, their username, and a temporary password. Participants will be required to set a new password the first time they access the app. Parents will be instructed to either (1) place a bookmark to the web app on their phone’s home screen or (2) install the web app to their phone, which will deliver a ‘native-like’ experience to participants.

Across the 6 weeks of the intervention, participants will receive SMS messages that contain feeding tips and links to the app, prompts to reengage with the app, and summaries of their activities on the app (e.g. summary of food offers tracked that week). In line with the evidence that personalised, tailored information supports behaviour change interventions, the SMS messages will be tailored to each participant based on child’s age, current feeding practises, feeding goals, and their own preferences (e.g. time of day, day of week, frequency).

### Control

Participants randomised to the control group will be wait-listed for the app and continue to receive any usual care. After a 6-week period, parents in the control group will complete follow-up measures and then be granted access to the app to the app.

### Participant compensation

Participants will be compensated for their time with a shopping gift voucher ($20AUD for each questionnaire completed—baseline and post-intervention—and $20AUD for participating in a post-intervention interview).

### Outcome measures

All measures will be collected at baseline and 6 weeks later at completion of the study. Outcomes will be assessed using online questionnaires, and app usage statistics will be collected throughout the study period.

#### Primary outcomes

The primary outcomes of this study relate to feasibility and acceptability of the intervention and the app platform: participant retention rate, intervention engagement, app usability, perceived ease in using the app, perceived usefulness of the app, and user satisfaction. Feasibility and acceptability outcomes will be assessed with data from the intervention group only. The measures for primary outcomes are provided in \* MERGEFORMAT Table 1.

**Table 1:** Feasibility and acceptability criteria and measures for the Fussy Eating Rescue pilot trial

Measures	Feasibility and acceptability criterion
Participant retention rate	At least 80% of enrolled participants complete the 6-weeks post-intervention questionnaire
Intervention engagement	At least 80% of intervention participants begin engaging with the app There are currently not standards of practice for what constitutes acceptable app engagement in digital health interventions for parents (58) App usage metrics will be explored and reported (e.g. session interval, session length, time in app, screen flow, retention, SMS received/read)
Usability of the app platform	Average System Usability Scale (SUS) [58] scores meet or exceed benchmark of 81 (the average score of the final usability tests before the pilot)
Perceived ease in using the app platform	Average mHealth App Usability Questionnaire (MAUQ) [59] scores are 80% of highest score possible
Perceived usefulness of the app platform	Average MAUQ ease of use, usefulness, and satisfaction subindex scores are 80% of highest score possible
Satisfaction with the app platform	Post-intervention interviews will explore further parents’ experiences using the app, including usability and acceptability, positive and negative aspects of the app, impact of the app on their life, and facilitators/barriers to using the app

#### Secondary outcomes

Secondary outcomes include parent feeding practises and children’s eating behaviours. Secondary outcomes will be measured using validated tools included in both the pre- and post-intervention questionnaires. Parent feeding practises will be captured using relevant subscales from the Child Feeding Practises Questionnaire (CFPQ, i.e. modelling) [60] and the FPSQ (family meal setting, persuasive feeding, reward for eating, and structured meal timing) [61]. The practise of repeated exposure will be measured with one question validated for this purpose [62]. Children’s food fussiness will be measured with the Food Fussiness subscale of the Child Eating Behaviour Questionnaire (CEBQ) [63]. Children’s specific food avoidance behaviours (e.g. eating slowly, hiding food, gagging) will be inventoried with the Meal Behaviour Questionnaire (MBQ) [64].

#### Demographics

On the baseline questionnaire, parents will report their age, highest level of education, relationship to their child (e.g. biological father, step-mother, other), family income (reported as: less than $40,000, $40,000–$49,000, $50,000–$59,000, $60,000–$69,000, $70,000–$79,000, $80,000–$89,000, $90,000–$99,000, $100,000–$149,000, and more than $150,000), household size, post code, employment status, country of birth, and the main languages spoken at home. Parents will also report their child’s age and gender, height, and weight.

### Analysis

Analysis of primary outcomes, related to feasibility and acceptability, will only be available from the intervention group and therefore cannot be blinded. Analysis of secondary outcomes, which will be collected from intervention and control participants, will be blinded. Data will be analysed with R [65] using RStudio software [66]. Frequencies and proportions will be calculated for categorical data; means and standard deviations will be calculated for continuous data; medians and IQRs will be calculated for ordinal data. Descriptive statistics derived from usage data will be used to characterise the engagement and acceptability of the intervention app. Specifically, building upon previous research conducted by the research team, an engagement index will be calculated from five subindices: click depth, loyalty, interaction, recency, and feedback [67, 68]. Although this pilot RCT will not be powered to detect intervention effects, the within-group mean differences and 95% CIs will be reported for changes in secondary intervention outcomes between baseline and 6 weeks. The standardised mean differences between intervention and control groups will be interpreted as 0.2 = small effect, 0.5 = moderate effect, 0.8 = large effect, and 1.2 = very large effect [69].

## Discussion

The current study is designed to evaluate the acceptability and feasibility of an mHealth intervention to improve the feeding practises of parents concerned for toddler fussy eating behaviours. This study will also assess a range of secondary outcomes including parent feeding practises, parent outcome expectations, parent capabilities, and children’s eating behaviours.

Responsive feeding practises have been shown to have beneficial impacts on children’s health and diet, especially for children with fussy eating behaviours [70]. However, parents concerned for fussy eating often respond with nonresponsive feeding practises that increase the severity and duration of fussy eating behaviours and have myriad negative impacts on children’s and parents physical and mental health [11, 18].

Previous studies have shown that are increasingly relying on digital platforms as sources for health information and support [71, 72]. However, no previous digital health interventions to improve the feeding practises of parents concerned for toddler fussy eating have been evaluated. A key factor in the success of digital health interventions is participant engagement [73] hence, it is critical to incorporate user-centred design methods and to evaluate feasibility and acceptability before proceeding to larger trials. We speculate that Fussy Eating Rescue intervention—developed via iterative design cycles that incorporated parent feedback—will be acceptable, feasible, and will promote responsive feeding practises among parents concerned for fussy eating. 

## Data Availability

The datasets generated and/or analysed during the current study are not publicly available but are available from the corresponding author on reasonable request.
